# Spatial-temporal characteristics of severe fever with thrombocytopenia syndrome and the relationship with meteorological factors from 2011 to 2018 in Zhejiang Province, China

**DOI:** 10.1371/journal.pntd.0008186

**Published:** 2020-04-07

**Authors:** Haocheng Wu, Chen Wu, Qinbao Lu, Zheyuan Ding, Ming Xue, Junfen Lin

**Affiliations:** 1 Zhejiang Province Center for Disease Control and Prevention, Hangzhou, Zhejiang Province, China; 2 Key Laboratory for Vaccine, Prevention and Control of Infectious Disease of Zhejiang Province, Hangzhou, Zhejiang Province, China; 3 Hangzhou Centre for Disease Control and Prevention, Hangzhou, Zhejiang, Province, China; Australian Red Cross Lifelood, AUSTRALIA

## Abstract

**Background:**

Zhejiang Province has the fifth-highest incidence of severe fever with thrombocytopenia syndrome (SFTS) in China. While the top four provinces are all located in northern and central China, only Zhejiang Province is located in the Yangtze River Delta region of southeast China. This study was undertaken to identify the epidemiological characteristics of SFTS in Zhejiang from 2011 to 2018.

**Methods:**

The epidemic data from SFTS cases in Zhejiang Province from January 2011 to December 2018 were obtained from the China Information Network System of Disease Prevention and Control. Meteorological data were collected from the China Meteorological Data Sharing Service System. A multivariate time series model was used to analyze the heterogeneity of spatial-temporal transmission of the disease. Random forest analysis was performed to detect the importance of meteorological factors and the dose-response association of the incidence of SFTS with these factors.

**Results:**

In total, 412 SFTS cases (49 fatal) were reported from January 2011 to December 2018 in Zhejiang Province, China. The number of SFTS cases and the number of affected counties increased year by year. The case fatality rate in Zhejiang Province was 11.89%, which was the highest in China. Elderly patients and farmers were the most affected. The total effect values of the autoregressive component, spatiotemporal component and endemic component of the model in all ranges were 0.4580, 0.0377 and 0.0137, respectively. There was obvious heterogeneity across counties for the mean values of the spatiotemporal component and the autoregressive component. The autoregressive component was obviously the main factor driving the occurrence of SFTS, followed by the spatiotemporal component. The importance scores of the monthly mean pressure, mean temperature, mean relative humidity, mean two-minute wind speed, duration of sunshine and precipitation were 10.64, 8.34, 8.16, 6.37, 5.35 and 2.81, respectively. The relationship between these factors and the incidence of SFTS is complicated and nonlinear. A suitable range of meteorological factors for this disease was also detected.

**Conclusions:**

The autoregressive and spatiotemporal components played an important role in driving the transmission of SFTS. Targeted preventive efforts should be made in different areas based on the main component contributing to the epidemic. For most areas, early measures several months ahead of the suitable season for the occurrence of SFTS should be implemented. The level of reporting and diagnosis of this disease should be further improved.

## Author summary

SFTS is listed as one of the nine most infectious diseases on the WHO priority list. The transmission route and the factors driving the epidemic were complex. Ninety percent of worldwide cases have been reported from China, and Zhejiang Province in China has a high case fatality rate and a high incidence of SFTS. In our study, we found that the numbers of affected counties increased year by year, and the interval from illness onset to confirmation in SFTS was still too long. Furthermore, spatial heterogeneity in the risk factors driving the transmission of SFTS was detected. In addition, the range of suitable weather values for the transmission of SFTS was identified. This suggests that targeted preventive efforts should be made in different areas based on the main factor contributing to the epidemic. In particular, early measures several months ahead of the suitable season for the occurrence of SFTS should be implemented. Enhancement of the level of reporting and diagnosis of this disease should strengthen ongoing research with the incorporation of more risk factors.

## Introduction

Severe fever with thrombocytopenia syndrome (SFTS) is an emerging hemorrhagic fever caused by the severe fever with thrombocytopenia syndrome virus (SFTSV), which is classified in the order Bunyavirales, family Phenuiviridae, and genus Phlebovirus[1–3]. SFTS was first confirmed among rural areas in China in 2009 and subsequently reported in North Korea in 2009, South Korea in 2012 and Japan in 2013 [4–7]. Furthermore, a cluster of cases of SFTS in 1996 was uncovered and reported in a new study. It has been suggested that the SFTSV has been circulating in China for more than 10 years prior to 2009[2]. An SFTSV-like virus called the Heartland virus was also isolated in the United States in 2012[8]. The main clinical symptoms of SFTS include fever, thrombocytopenia, leukocytopenia, gastrointestinal symptoms, neurological symptoms and bleeding tendency, as well as less specific clinical manifestations [9,10]. The average case fatality rate typically varies from 8% to 12%[2,11], but it can reach 32.6%[12,13].

The SFTSV is believed to be transmitted through tick bites, direct contact with SFTS patient secretions or blood, and probable aerosol transmission [14–18]. The SFTSV can be detected in humans, various domestic animals and ticks, and the animals may be a reservoir host in maintaining the life cycle of the SFTSV in nature [9]. Previous studies have reported that meteorological factors may influence the ecology of the SFTSV by affecting tick growth dynamics, tick-human interactions and virus replication[19]. Meteorological factors, including temperature, relative humidity, and precipitation, and environmental factors, such as land cover, normalized difference vegetation index (NDVI), and the density of cattle, have been related to the occurrence of SFTS[19]. However, the relationship between SFTS epidemics and factors such as pressure, wind speed and duration of sunshine has seldom been reported[20–23].

SFTS was listed as one of the nine most infectious diseases on the WHO priority list in 2017[2]. This disease is a severe public health issue in mainland China, and approximately 90% of worldwide cases have been reported from China [4]. From 2011–2016, a total of 5360 laboratory-confirmed SFTS cases and 343 deaths were reported in China [24]. The number of annual cases and affected counties in China have increased year by year [24]. The five provinces with the highest SFTS incidence are Henan, Shangdong, Hubei, Anhui and Zhejiang [24]. While the top four provinces are all located in northern and central China, Zhejiang, ranked fifth-highest in SFTS incidence, is located in the Yangtze River Delta region of southeast China. The objective of this study was to identify the epidemiological characteristics of SFTS in Zhejiang from 2011 to 2018. The heterogeneity of spatial-temporal transmission of this disease was analyzed to identify the main components driving SFTS transmission at the county level. Furthermore, the dose-response association of the incidence of SFTS with meteorological factors was identified to find the range of suitable weather values for the transmission of SFTS and to implement control measures at an early time.

## Materials and methods

### Ethical review

This study was reviewed and approved by the Ethics Committee of the Zhejiang Provincial Center for Disease Control and Prevention. All the data of the individuals were kept confidential as requested. According to the law of the People’s Republic of China on the prevention and treatment of infectious diseases, demographic and disease information on SFTS was forced to be reported, so written consent was not necessary. The use of verbal consent was approved by our Ethics Committee. Verbal informed consent was obtained from all patients prior to diagnosis and to reporting to the China Information Network System of Disease Prevention and Control. However, verbal consent could not be documented. All the methods employed in the study were in accordance with the applicable guidelines and regulations.

### Profile of Zhejiang Province

Zhejiang Province is located in southeast China between longitudes 118°E-123°E and latitudes 27°N-32°N. There are two subprovincial cities (Hangzhou and Ningbo) and nine prefecture-level cities, including Wenzhou, Huzhou, Jiaxing, Shaoxing, Jinhua, Zhoushan, Quzhou, Taizhou and Lishui, which cover 90 counties.

### Data collection

Any human SFTS case diagnosed in a hospital must be reported through the China Information Network System of Disease Prevention and Control by the medical staff. The data on the SFTS cases in Zhejiang Province from January 2011 to December 2018 were obtained from this network system. Individual information on cases and deaths was imported, and surveillance information, including demographic characteristics and geographic and temporal distributions, was computed by the system. Reported cases were defined according to the national guideline for prevention and control for SFTS issued by the Chinese Ministry of Health in 2010[19]. Meteorological data, including monthly mean temperature, monthly precipitation, monthly mean pressure, monthly mean relative humidity, monthly mean two-minute wind speed and monthly duration of sunshine, were collected from the China Meteorological Data Sharing Service System (http://data.cma.cn/).

### Joinpoint regression

Joinpoint regression was used to describe continuous changes in the incidence trends, and the grid-search method was used to fit the regression function, with unknown joinpoints assuming constant variance and uncorrelated errors[25]. The joinpoint regression model for observations (*x*_1_,*y*_1_),…,(*x*_*n*_,*y*_*n*_), where *x*_1_≤…≤*x*_*n*_ without a loss of generality, may be written as

$$E[y/x]=\beta_{0}+\beta_{1}+\delta_{1}{\left(x-\tau_{1}\right)}^{+}+\ldots +\delta_{k}{\left(x-\tau_{k}\right)}^{+},$$ (1)

where y is the outcome of interest, x is the time variable, *τ*_*k*_'*s* are the unknown joinpoints, and *α*^+^ = *α* for *α*>0 and 0 otherwise. The approximate permutation test was used to find the number of significant joinpoints; each p-value was found using Monte Carlo methods, and the overall asymptotic significance level was maintained through Bonferroni correction. The null hypothesis is 0 joinpoints. The objective indicator was the annual percent change (APC) of each period segment, which was estimated according to the following formula:

$$APCi=[(Exp(\beta_{i})-1]\times 100,$$ (2)

where *β*_*i*_ represents the slope of the period segment [25,26].

### Random forest analysis

Random forests are widely used for data prediction and interpretation purposes [27,28]. Random forests are made up of many single trees. The latter are fit to bootstrapped data, while splits are found in random sets of variables. Predictions are given by averaged values or majority votes of each tree’s prediction. ‘Out-of-bag’ (OOB) samples are used for an unbiased estimate of the prediction error. The permutation importance measure for variables is computed by the difference of a tree’s OOB error before and after random permutation of a predictor variable. The mean value of all trees is the actual importance of a variable. If the variable is of relevance for prediction in the forest, the error is supposed to increase by permutation. The increase in the mean of squared residuals (%IncMSE) is usually used to measure the importance of the variable[29]. The measures of computing the importance can be divided into five steps:

1. Compute the OOB error of a tree.
2. Randomly assign each observation to the child nodes.
3. Recompute the OOB error of the tree (following step 2).
4. Compute the difference between the original and recomputed OOB errors.
5. Repeat steps 1–4 for each tree and use the average difference over all trees as the overall importance score.

### Multivariate time series model

The multivariate time series model established by Held and Paul was designed for spatially and temporally aggregated surveillance data. *Y*_*i*,*t*_ is the disease count in region *i* = 1,…,*I* at time *t* = 1,…,*T*. The count *y*_*i*,*t*_ is formally assumed to follow a negative binomial distribution *Y*_*it*_/*Y*_*i*,*t*−1_~*NegBin*(*μ*_*it*_,*ψ*) *i* = 1,…,*I*,*t* = 1,…,*T*, with an additively decomposed mean

$$\mu_{it}=\nu_{it}e_{it}+\lambda_{it}Y_{i,t-1}+\phi_{it}\sum_{j\neq i}\omega_{ji}Y_{j,t-1},$$ (3)

where *ψ* is an overdispersion parameter such that the conditional variance of *Y*_*it*_ is *μ*_*it*_(1+Ψ*μ*_*it*_). The Poisson distribution is the result of the special case where *ψ* = 0. The first component *ν*_*it*_*e*_*it*_ represents the endemic component, which captures exogenous factors such as population, sociodemographic variables, long-term trends, seasonality, and climate. The endemic mean is proportional to an offset of known expected counts *e*_*it*_, typically reflecting the population at risk. As a district-specific measure of disease incidence, the population fraction *e*_*it*_ is included as a multiplicative offset. Here, the population at the county level as a multiplicative offset was incorporated into the endemic component. The next two components are observation-driven epidemic components. The second component *λ*_*it*_*Y*_*i*,*t*−1_ is an autoregression on the number of cases at the previous time point. The third component $\phi_{it}\sum_{j\neq i}\omega_{ji}Y_{j,t-1}$ denotes the spatiotemporal characteristics capturing the transmission from the other units. Each parameter *ν*_*it*_,*λ*_*it*_, and *ϕ*_*it*_ is a linear predictor of the form

$$\log(\cdot it)=\alpha^{\left(\cdot \right)}+b_{i}^{\left(\cdot \right)}+\beta^{{\left(\cdot \right)}^{T}}z_{it}^{\left(\cdot \right)},$$ (4)

where “.” is one of *ν*,*λ*,*ϕ*; *α*^(.)^ are the intercepts; and $b_{i}^{\left(\cdot \right)}$ denotes random effects, which account for the heterogeneity between districts. $z_{it}^{\left(\cdot \right)}$ are exogenous covariates, including time effects, and $\beta^{{\left(.\right)}^{T}}$ denotes the coefficient of $z_{it}^{\left(\cdot \right)}$.

*ω*_*ji*_ is the spatial contiguity weights matrix, which describes the strength of transmission from region *j* to region *i*. There are usually three models of neighborhood weights, including the first-order neighborhood model, the power law model and the second-order neighborhood model[30,31]. The first-order neighborhood model assumes that an epidemic can only arrive from directly adjacent districts and that all districts have the same coefficient for importing cases from neighboring districts. The *k*th-order neighborhood model and the power law model were used to account for long-range case transmission; in other words, the idea that the disease can be transmitted across the county. The variance components are estimated by maximizing the approximated marginal likelihood obtained via a Laplace approximation.

### Statistical analysis

The joinpoint regression model was used to examine the trend of the incidence of SFTS from 2011 to 2018 with Joinpoint software (version 4.5.0.1). The multivariate time series model and random forest analysis were run by R software (version 3.3.1). A *P* value less than 0.05 represented statistical significance for all the tests.

## Results

### General epidemiological characteristics

**Fig 1:**
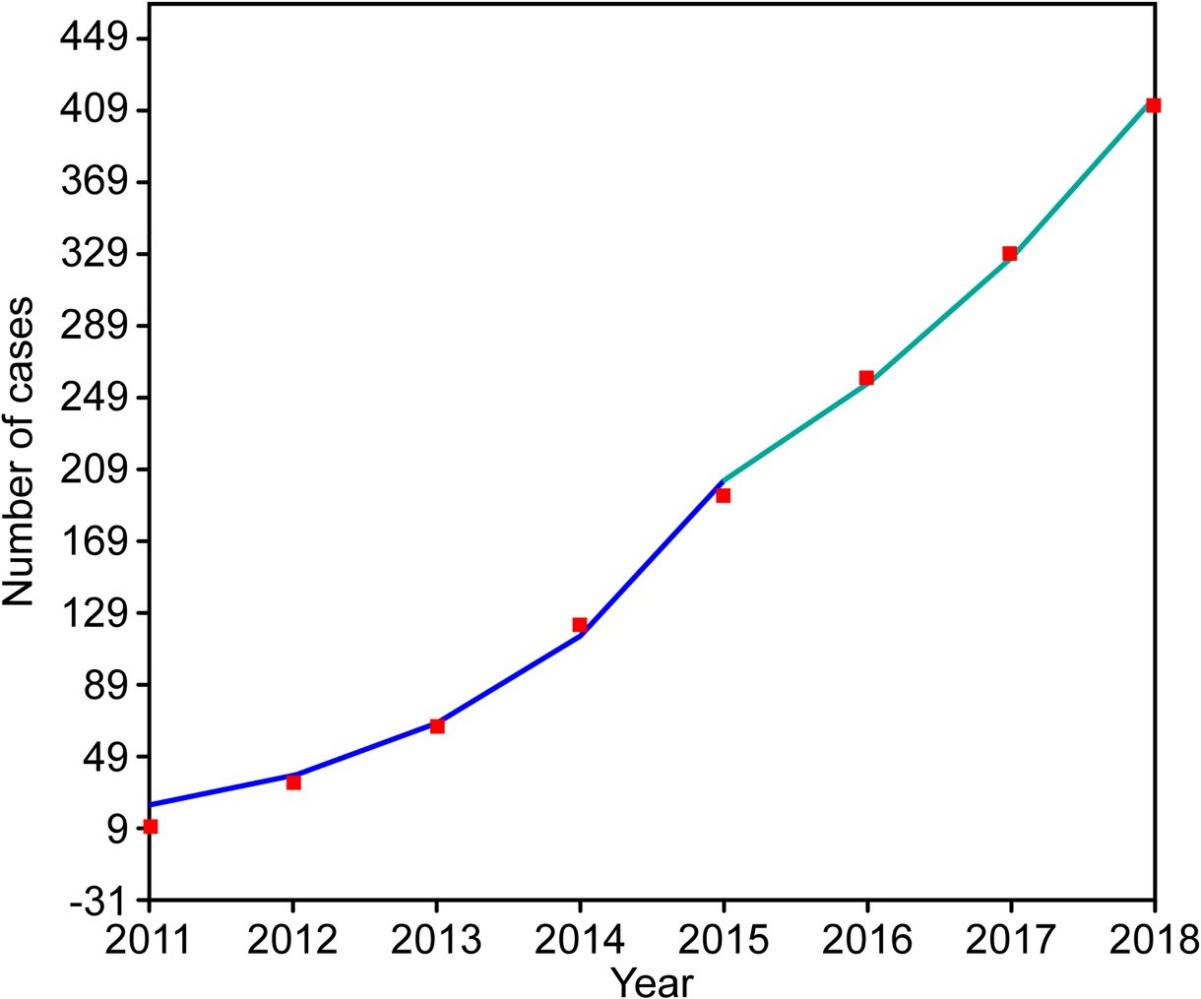
Trend of the incidence of SFTS between 2011 and 2018 shown by the joinpoint regression. The red squares denote the yearly cumulative cases, and the blue line is the slope of the APC, the annual percent change.

**Fig 2:**
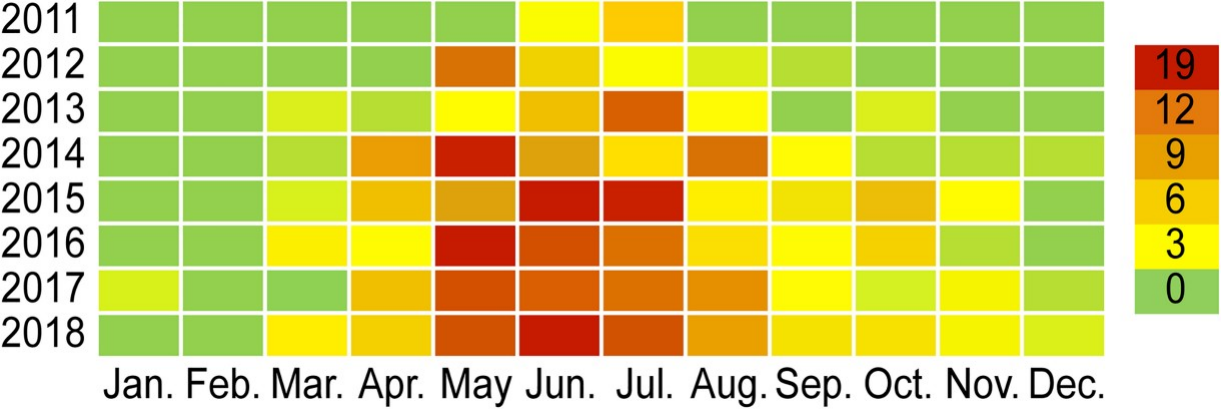
Heat map of the seasonal distribution of SFTS cases in Zhejiang Province, China, 2011–2018.

A total of 412 SFTS cases (49 fatal) were reported from January 2011 to December 2018 in Zhejiang Province, China. The average case fatality rate was 11.89%. There were 9, 25, 31, 57, 72, 66, 69 and 83 cases identified in each year. Meanwhile, 0, 4, 4, 10, 8, 6, 8 and 9 fatalities occurred each year. The increasing trend of annual cumulative case numbers varied across different years. There was 1 joinpoint in the final model (*P* = 0.003). The annual percent change (APC) of cumulative incidence was 74.7% (95%CI: 52.8% to 99.6%) from 2011 to 2015, and the APC was 27.1% (95%CI: 23.9% to 30.3%) after 2016. These two values were all significantly different from zero at the alpha = 0.05 level (test statistics = 13.3 & 30.4, respectively, *P*<0.001). This result indicates a monotonic incremental trend in the incidence. However, the APC from 2016 to 2018 is smaller than that before 2015, suggesting that the number of cases was relatively stable among these years (Fig 1). The mean time from illness onset to confirmation for SFTS cases among all periods was 8 days; for the individual years from 2011 to 2018, these numbers were 13 days, 19 days, 8 days, 9 days, 6 days, 5 days, 6 days and 7 days. Obvious seasonality was present for the occurrence of SFTS across the eight-year period. There was an apparent incidence peak every year in spring-summer; the cases reported from May to July accounted for 62.86% of the total cases. Relatively fewer cases were reported in January, February and December compared to the other months (Fig 2).

The majority of SFTS patients were farmers (66.75%), followed by houseworkers (22.82%). Among all patients, the proportion of female SFTS patients (52.18%) was approximately equal to that of male patients (47.82%), and the total female-to-male ratio was 1.09. The median age of the SFTS patients across the eight years was 66 years. The three age groups with the most reported cases were 60–69 years (35.52%), 70–79 years (25.49%) and 50–59 years (18.93%).

### Multivariate time series analysis

**Fig 3:**
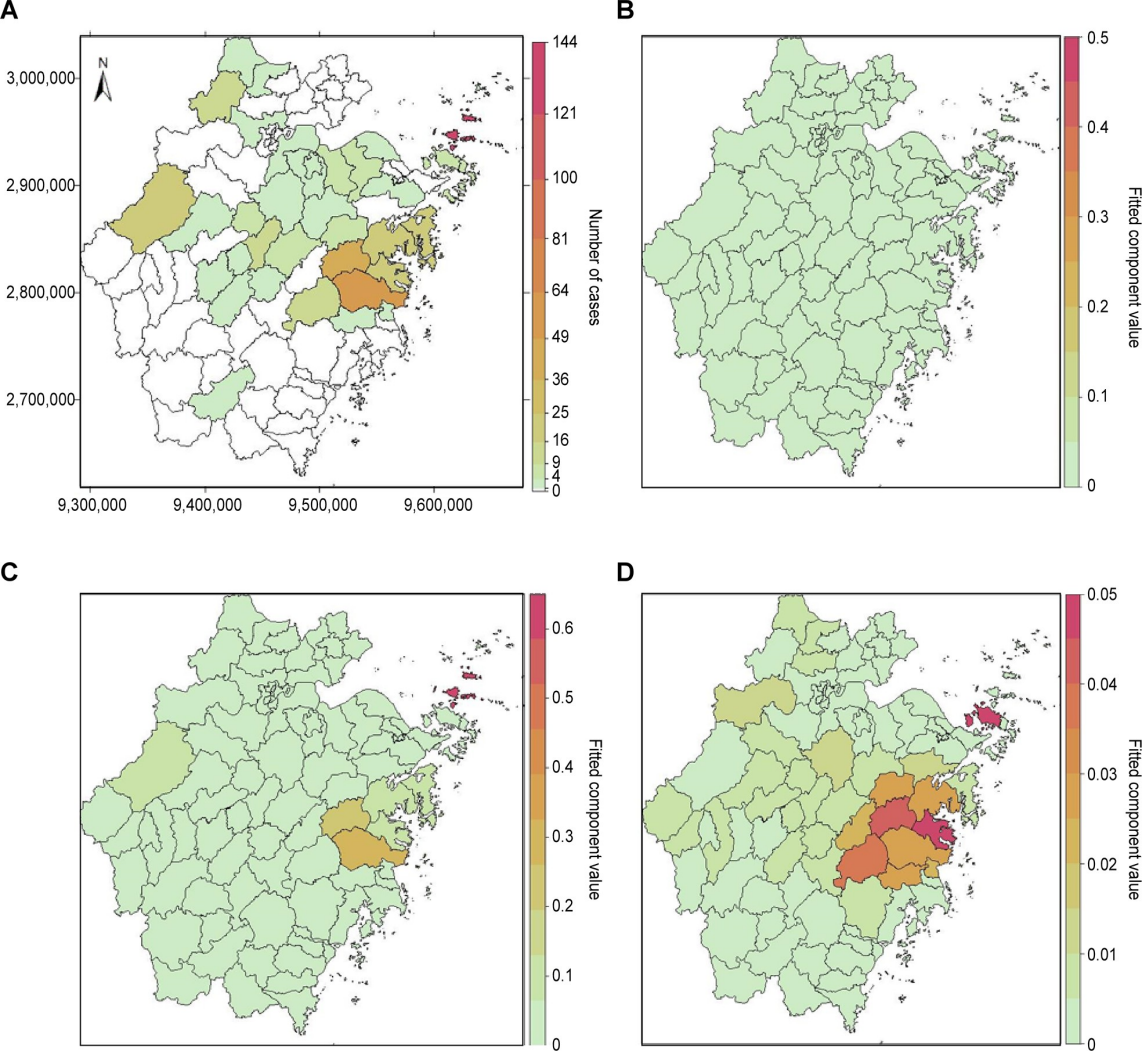
The district-specific total incidence and fitted component of SFTS in Zhejiang Province, China, 2011–2018. These maps were created by R software (version 3.3.1, http://www.r-project.org/). (A) The total incidence of SFTS from 2011 to 2018. (B) The endemic component at the county level. (C) The autoregressive component at the county level. (D) The spatiotemporal component at the county level.

During 2011–2018, 37 counties (41.11%), including 118 towns, were affected. The numbers of affected counties increased year by year (4, 5, 7, 15, 13, 15, 19 and 24). Approximately 74% of SFTS cases were circulated in seven counties: 130 cases were reported in Daishan County, 62 cases in Linhai County, 44 cases in Tiantai County, 21 cases in Ninghai County, 17 cases in Xiangshan County, 16 cases in Chunan County and 16 cases in Sanmen County (Fig 3).

To determine the heterogeneity of spatial-temporal transmission, a multivariate time series model was constructed based on monthly data from 2011 to 2018. First, we built two models to select the distribution of the incidence. We assumed that the incidence would follow a negative binomial distribution in one model and the Poisson distribution in the other, with the first-order neighborhood regarded as the neighborhood weight by default. The AICs of these two models were 2256.43 and 2469.81, which suggested that the assumption of a negative binomial distribution of the incidence would be better for modeling. Second, based on the assumption of a negative binomial distribution of the incidence, we built three models to ascertain the spatial contiguity weights matrix. The first-order neighborhood model, the power law model and the second-order neighborhood model were compared. The AICs of the three models were 2256.43, 2258.48 and 2259.07. This indicated that the first-order neighborhood model was slightly better than the other two models.

The total effect values of the autoregressive component, spatiotemporal component and endemic component in all ranges were 0.4580, 0.0377 and 0.0137, respectively. The autoregressive component was more considerable than the other two components. This suggested that the occurrence of SFTS in Zhejiang Province, China, was majorly influenced by the autoregressive factor instead of the endemic component and spatiotemporal component.

There was obvious heterogeneity across counties for the mean values of the spatiotemporal component and the autoregressive component from the maps (Fig 3). However, the mean values of endemic components in different counties were not different. For the spatiotemporal component, there was interesting heterogeneity across the eleven counties with the highest incidence of SFTS. The top eleven counties and their component values, with cumulative incidences greater than 8, were Sanmen (0.0496), Dinghai (0.0494), Tiantai (0.0449), Xianju (0.0425), Ninghai (0.0318), Linhai (0.0302), Xiangshan (0.0083), Yiwu (0.0064), Daishan (0.0032), Anji (0.0016) and Chun’an (0.004). The top six counties with mean spatiotemporal component values greater than 0.03 were located in the eastern parts of Zhejiang Province. These counties were close to each other except Dinghai, which is an island in Zhoushan city. Furthermore, there was significant spatial heterogeneity among the districts for the autoregressive component. The county with the highest mean value of the autoregressive component was Daishan (0.6267), which is located on the northeastern island of the province. The other top 10 counties with high fitted values of the autoregressive component were Linhai (0.2941), Tiantai (0.2073), Ninghai (0.1012), Xiangshan (0.0820), Chun’an (0.0771), Sanmen (0.0771), Anji (0.0627), Yiwu (0.0578), Xianju (0.0530) and Dinghai (0.0382) (Table 1).

**Table 1:** The mean values of the components of the top eleven counties with the highest incidence of SFTS.

county	endemic component	county	autoregressive component	county	spatiotemporal component
Chun’an	0.0182	Daishan	0.6267	Sanmen	0.0496
Xiangshan	0.0182	Linhai	0.2941	Dinghai	0.0494
Ninghai	0.0182	Tiantai	0.2073	Tiantai	0.0449
Anji	0.0182	Ninghai	0.1012	Xianju	0.0425
Yiwu	0.0182	Xiangshan	0.0820	Ninghai	0.0318
Daishan	0.0182	Chun’an	0.0771	Linhai	0.0302
Sanmen	0.0182	Sanmen	0.0771	Xiangshan	0.0083
Tiantai	0.0182	Anji	0.0627	Yiwu	0.0064
Xianju	0.0182	Yiwu	0.0578	Daishan	0.0032
Linhai	0.0182	Xianju	0.0530	Anji	0.0016
Dinghai	0.0182	Dinghai	0.0382	Chun’an	0.0004

**Fig 4:**
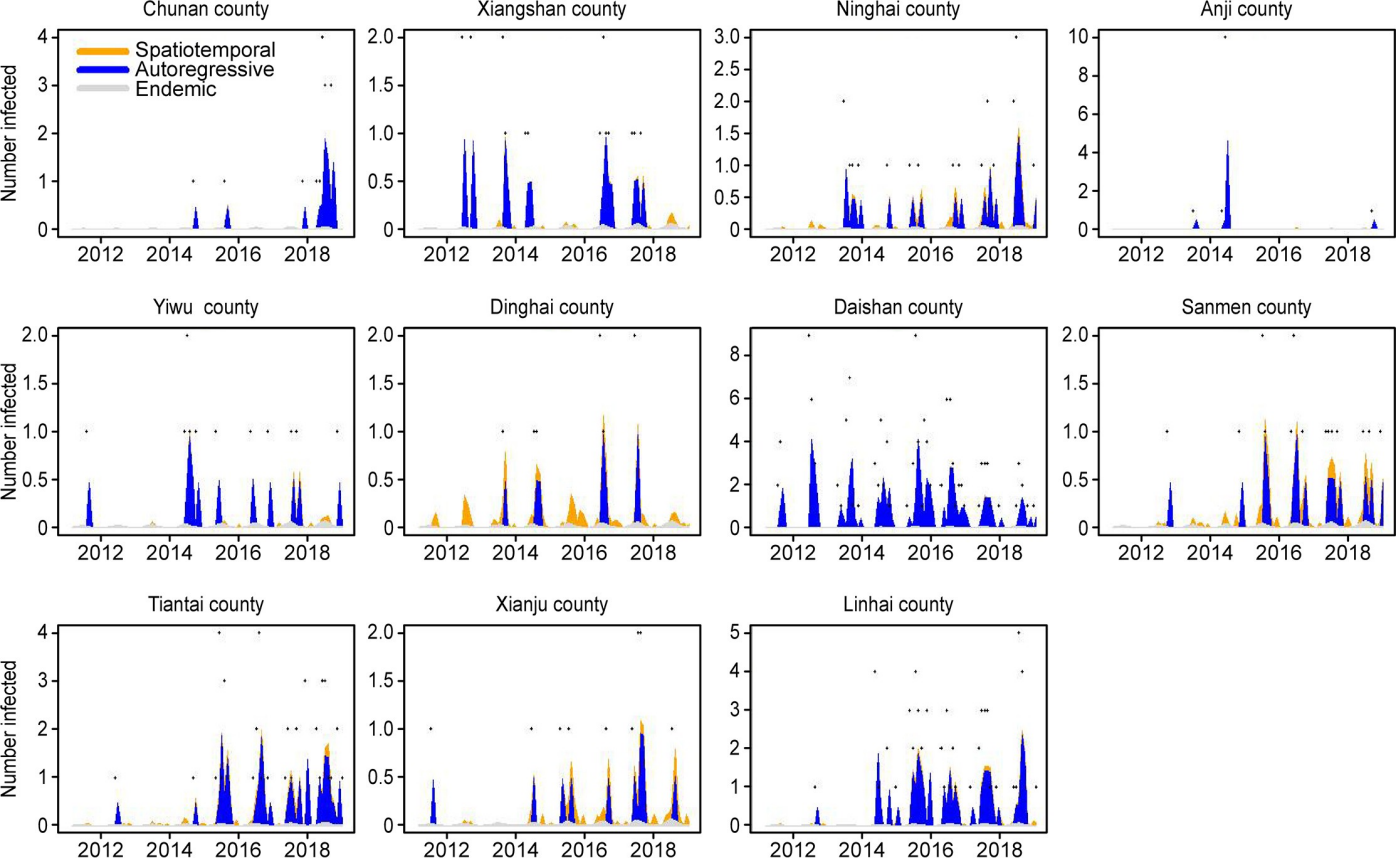
Fitted components in the multivariate time series model for the counties with more than 8 cases during the past eight years. The black dots represent the monthly incidence, the light gray area shows the endemic component, the blue area shows the autoregressive component, and the yellow area corresponds to the spatiotemporal component.

To identify the time-varying importance of the three components in the high incidence areas (>8 cases over eight years), we plotted the components along with the observed counts. The relative contributions of the three components in driving the prevalence of SFTS over time are displayed in these figures (Fig 4), and seasonal characteristics can be seen at the same time.

There were considerable differences in the time-varying effects of the three components among the aforementioned high incidence areas. The seasonality was apparent, especially for the autoregressive and spatiotemporal components. Similar to the results mentioned above, the effects of the endemic components for all counties were slight across all periods. The autoregressive component was obviously the main factor driving the occurrence of SFTS for all these counties. The autoregressive influence was also clearly observed in the incidence peak. This disease was first identified in Daishan, Yiwu and Xianju in 2011 and was then discovered in other counties. The counties of Chun’an, Anji and Daishan were almost exclusively influenced by the autoregressive factor over the whole period. Xiangshan and Ninghai, which are part of Ningbo city, are close to each other. The epidemic in Xiangshan occurred earlier than that in Ninghai, and the spatiotemporal component can be identified in Ninghai from 2012–2013, at the beginning of the epidemic in this county. After that, the spatiotemporal component can be detected between the two counties for the remainder of the period. Interestingly, Dinghai, an island of Zhoushan city, was continually influenced by spatiotemporal factors. Furthermore, the obvious spatiotemporal component can also be observed in Sanmen, Tiantai and Xianju after the first case was reported. These three counties are adjacent, and the spatiotemporal component was another important driving factor for them in addition to the autoregressive component. Linhai, which is near the three counties mentioned above, was mainly affected by autoregressive factors and slightly influenced by the spatiotemporal component.

### The relationship with meteorological factors

The median monthly mean temperature, monthly precipitation, monthly mean pressure, monthly mean relative humidity, monthly mean 2-minute wind speed and monthly duration of sunshine were 18.98°C, 120.22 mm, 1011.97 hPa, 75.75%, 1.82 m/s and 123.38 h, respectively (Table 2).

**Table 2:** The meteorological factors in the study area from 2011 to 2018.

Variables	Min	Median	Mean	Max
**monthly mean temperature(°C)**	3	18.98	18.21	30
**monthly precipitation(mm)**	16	120.22	138.93	326
**monthly mean pressure(hPa)**	999	1011.97	1011.85	1025
**monthly mean relative humidity (%)**	60	75.75	75.44	89
**monthly mean 2-minute wind speed(m/s)**	1	1.82	1.82	3
**monthly duration of sunshine(h)**	41	123.38	131.22	275

**Fig 5:**
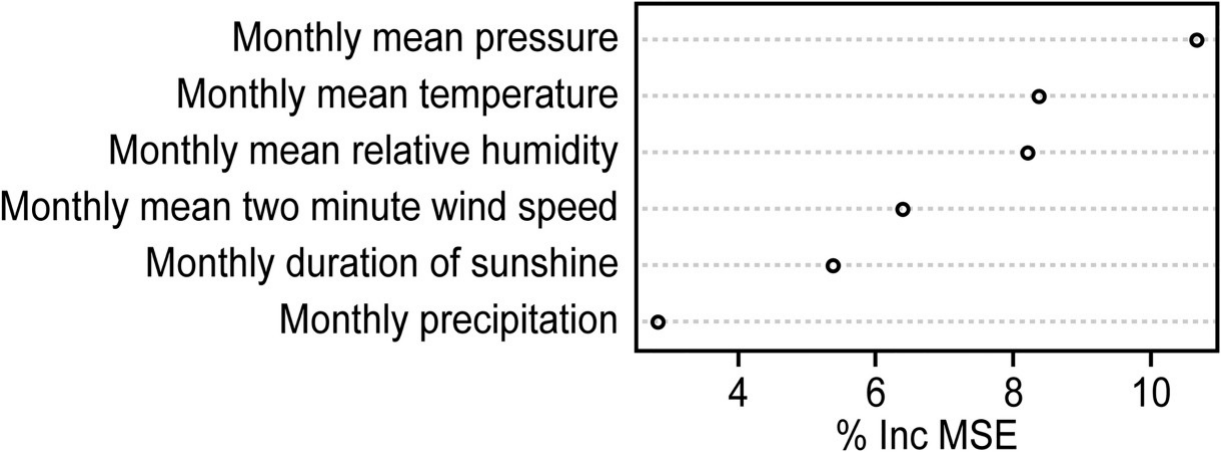
Importance of the climate variables on SFTS incidence.

According to Fig 5, the most important climate factor for SFTS was the monthly mean pressure, with a %IncMSE greater than 10, followed by monthly mean temperature, monthly mean relative humidity, monthly mean two-minute wind speed and monthly duration of sunshine. The %IncMSE of these meteorological factors were 8.34, 8.16, 6.37 and 5.35, respectively. Finally, the least important factor was the monthly precipitation, with an %IncMSE less than 3 (Fig 5).

**Fig 6:**
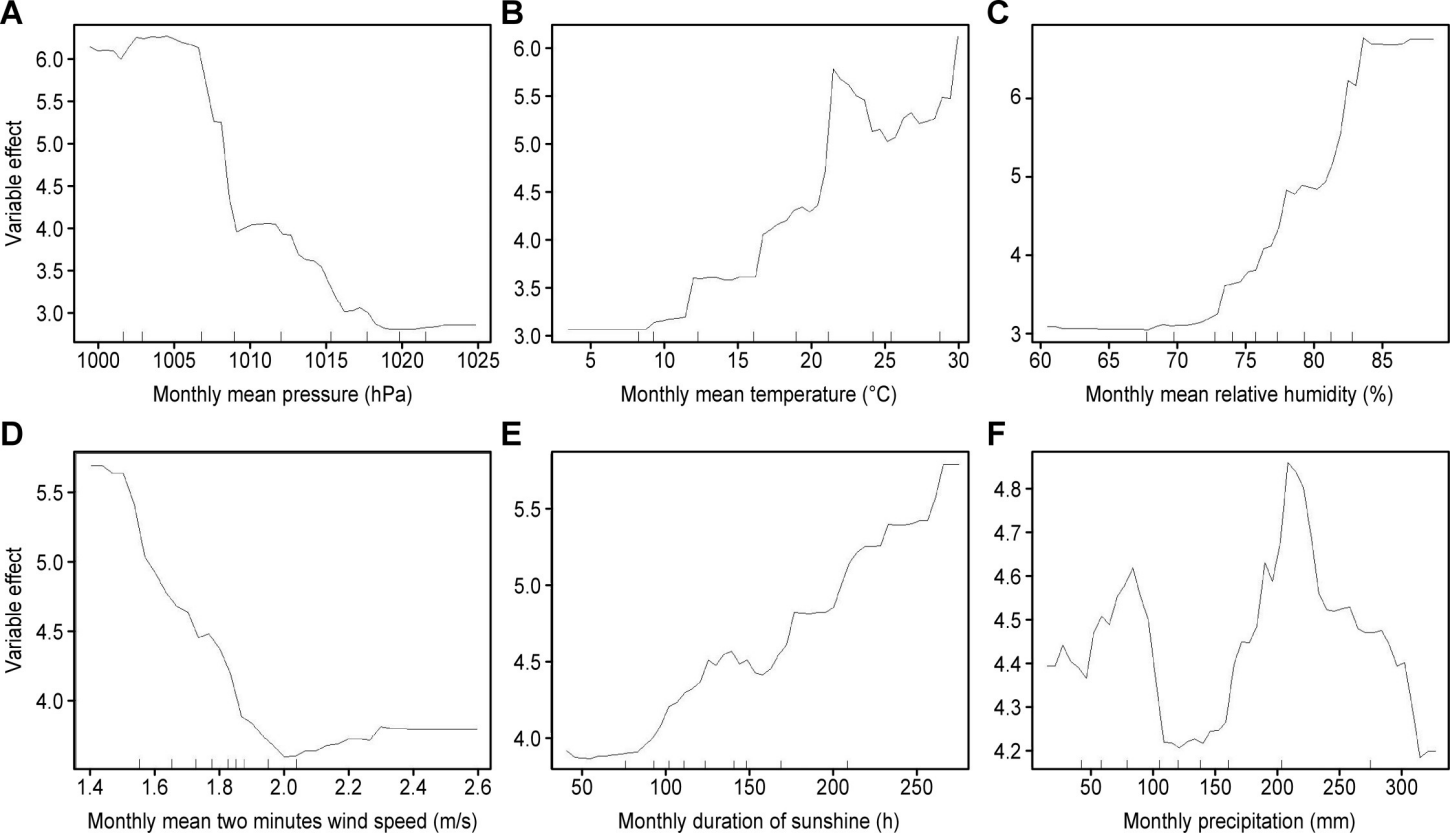
The relationship between meteorological factors and SFTS. A. Response curves for monthly mean pressure. B. Response curves for monthly mean temperature. C. Response curves for relative humidity. D. Response curves for monthly mean two-minute wind speed. E. Response curves for monthly duration sunshine. F. Response curves for monthly precipitation.

Furthermore, the relationship between the meteorological factors and the occurrence of SFTS was analyzed. The relationship between these factors and the incidence of SFTS was nonlinear (Fig 6). For monthly mean pressure, a single effect peak was found at a mean pressure below 1006.51 hPa and the mean effect value during the peak was 6.17. After that, the effect value decreased rapidly to a low level. For monthly mean temperature, two effect peaks were identified. This suggested that the suitable range of mean temperatures was from 21.5°C to 23.6°C and from 28.9°C to 30.0°C, with a mean effect greater than 5.64. On the whole, the risk of the occurrence of SFTS in warm weather with temperatures greater than 20°C was higher than that in cold weather. The effect of monthly relative humidity and the duration of sunshine showed a similar characteristic: the risk of the occurrence of SFTS increased with the growth of the relative humidity and the duration of sunshine. When the monthly mean relative humidity was greater than 81.39% and the monthly duration of sunshine was greater than 209.65 h, the effect values were continuously higher than 5. For the monthly mean two-minute wind speed, a short effect peak was observed from 1.40 m/s to 1.53 m/s, with an effect value greater than 5.41. Then, the effect value decreased gradually to the lowest level when the wind speed reached 2.00 m/s and slightly rose thereafter. There were also two effect peaks for monthly precipitation. The effect peak with precipitation from 189.42 mm to 232.74 mm was slightly higher than that from 71.86 mm to 90.42 mm. However, the highest effect value of precipitation was only 4.86, less than the mean peak value of the first five meteorological factors.

## Discussion

According to our research, the number of SFTS cases and the number of affected counties increased year by year. Zhejiang Province is one of the top five provinces with the highest incidence rate of SFTS; furthermore, its fatality rate is also higher than the overall average for China[24]. SFTS epidemics remain an important public health threat in Zhejiang Province, China. The reason for the increasing trend in the spatial-temporal dimension may be attributed to the improvement in diagnostic and detection capability of medical institutions due to the high fatality rate[24]. All municipal centers for disease control and prevention in Zhejiang Province established detection methods for SFTSV starting in 2014; correspondingly, the incidence and the number of affected counties began simultaneously increasing that year. Another reason may be that the SFTSV spread to more areas as a result of human, small mammal and tick migration[24]. In particular, migratory wild birds infected by the SFTSV or carrying SFTSV-infected ticks might play an important role in the long-distance spread of SFTSV [32,33]. A serious problem was found: the case fatality rate in Zhejiang Province was the highest among China. Previous studies have indicated that a long interval from illness onset to confirmation or a misdiagnosis of SFTS as hemorrhagic fever with renal syndrome (HFRS) is associated with an increased risk of fatality[34,35]. In our study, we found that the mean interval was more than one week, with the interval for a few cases approaching one month. This long interval from illness onset to diagnosis would lead to delay in the key period for treatment and increase the risk of fatality outcomes. The occupation and age distributions of SFTS patients were consistent with the results of previous studies[4,24]. Farmers accounted for the highest proportion of all occupations. These people are expected to participate more frequently in outdoor activities, which would increase exposure to ticks[24]. Furthermore, elderly patients constituted the most prominent age group. The high probability of severe disease or even death from SFTSV infection is the main reason that a greater number of elderly patients were identified[24,36]. Additionally, the demographic features of elderly residents in areas where SFTS cases occurred may be another factor influencing the age distribution[24].

To our knowledge, there are multiple routes of transmission of this disease, and the relationship with meteorological or environmental factors is also complicated. The different models of these factors can lead to different patterns of disease transmission and cause variations in the characteristics of the epidemics among the counties. Based on the multivariate time series model, the obvious spatial heterogeneity in the components driving SFTS transmission was captured. There were no obvious differences in the endemic component at the county level. This suggests that the factors including climatological changes and genetic susceptibility may be similar among the counties. The endemic risk of the occurrence of SFTS cases for all the counties also showed no difference. However, the number of reported incidences of SFTS cases varied widely at the county level, and no cases were identified among nearly 60% of the counties. The reason for the discrepancy in the risk and the number of cases at the county level may be that the data were collected from a passive surveillance system. Meanwhile, some counties possessed relatively low diagnostic levels, so a large number of unreported cases would increase the uncertainty of the estimation of risk. For most of the high incidence counties, the autoregressive component played a key role in driving the transmission of SFTS. This means that the epidemic effect in the previous season continuously contributed to the later peaks. The results also suggest that efforts to prevent and control SFTS, including decreasing tick density and implementing case management and health education, were not sufficiently powerful. Therefore, the epidemics circulated over time in these endemic areas and even led to local outbreaks at Chun’an in 2018 and Anji in 2014[37]. Daishan, Chun’an and Anji, the three counties located in the northeast or the northwest edge of Zhejiang Province, were almost solely affected by the autoregressive component, whereas the epidemics in other counties with high incidences were attributed to the spatiotemporal and autoregressive components at the same time. The epidemic in Xiangshan occurred earlier than that in Ninghai. After the occurrence of SFTS in Xiangshan, the spatiotemporal component could be detected in its neighboring county, Ninghai. This suggests that the SFTSV would probably be transmitted from Xiangshan to Ninghai and then circulated within Ninghai as an endemic disease. Additionally, the epidemics in these two counties continually affected each other. In contrast to those of the other counties, the epidemic in Dinghai was mainly driven by the spatiotemporal component, which indicates that the cases in this area may have been imported from neighboring areas, especially Daishan, an area with the highest incidence in Zhejiang Province. Given the traffic convenience between these two islands (only a 30-minute voyage), individuals who work in Daishan but live and seek medical advice in Dinghai were identified. Other areas with high spatiotemporal component value clusters should be noted. These areas are located in the eastern part of Zhejiang Province, including Sanmen, Tiantai, Xianju, Linhai and their surrounding regions, such as Huangyan, Xinchang and Pan’an. This result suggests that the cross-regional importing of SFTS played an important role in spreading the disease among the above counties. The reason for the spatial expansion of the disease may be attributed to border-crossing transportation of domestic animals that carried ticks and led to the infection. Another possibility could be that individuals who did farm work in neighboring areas with high incidences but lived and sought medical advice in their hometowns were infected with SFTSV. The situation in Yiwu and its neighboring counties, which are located in the central part of Zhejiang Province, was similar to that in the eastern areas.

As a disease of natural focus, the occurrence of SFTS depends mainly on meteorological factors that determine the distribution of vectors (ticks) and animal hosts[20]. To identify the importance of various kinds of these factors and the ranges of suitable weather values for the transmission of SFTS, the relationship between the occurrence of SFTS and meteorological factors was computed. According to the results of the importance scores of the meteorological factors, the incidence of SFTS was very sensitive and negatively related to atmospheric pressure. A possible explanation is that lower pressure may lead to a longer period of questing and a lower mortality rate in ticks. This may contribute to the high feeding success of nymphs on their natural hosts and increase the chance of human exposure to ticks[21]. Consistent with previous studies[19,22–23,38], temperature and relative humidity also played important roles in the dynamics of SFTS. The optimal ranges of relative humidity and temperature in our study were similar to the results of other papers[19,38]. The metabolic rate, growth rate and fecundity of ticks accelerate in warm and humid weather. Furthermore, temperature can directly influence the replication and dissemination of the SFTSV. In addition, humans as well as animals are more likely to spend time on outdoor activities at temperatures greater than 20°C, which enhances the probability of exposure to ticks. These possibilities function together in explaining the association between the SFTS epidemic and relative humidity and temperature[20,38]. As noted in a previous study, wind speed also affects the growth and reproduction of ticks[21]. A negative relation between the incidence of SFTS and wind speed was also found in our study. Continuous wind may increase the dispersal of carbon dioxide, which is an attractant for Ixodid ticks, but hasten forest air replacement by dryer air, which results in an increase in tick mortality[21]. Therefore, this may be the reason that the incidence decreased gradually as the wind speed rose. The duration of sunshine also showed a positive relation with the incidence of SFTS as well as with temperature, but the importance score was relatively lower than those of the first four meteorological factors. Because the time series of temperature and sunshine are in phase, the effect of sunshine may be an indirect function of temperature. In our study, the occurrence of SFTS was relatively insensitive to precipitation. The association between the incidence of SFTS and precipitation may be indirect and confounded by other factors.

Based on the high patient fatality rate, the heterogeneity of the components driving SFTS transmission and the suitable range of meteorological factors for this disease, targeted preventive efforts are needed in different areas. To reduce the fatality rate of SFTS, early diagnosis of this disease is necessary. Because of the homogeneity of the endemic risk, hospitals in all counties should improve the ability to detection SFTS patients. Especially for areas without case identification, timely diagnosis is also helpful to avoid clustering, which is attributed to human-to-human transmission. For most counties with a high incidence influenced mainly by the autoregressive component, early implementation several months ahead of the peak may effectively decrease the number of cases of SFTS in the peak season. Measures should be strengthened to reduce tick density ahead of the suitable season for the circulation of SFTS. This is crucial for controlling the endemic spread of the disease in these areas. For Sanmen, Dinghai, Tiantai, Xianju Ninghai and Linhai counties, which are affected by the spatiotemporal component as well as autoregressive factors, it is recommended to control the epidemic in their own district and neighboring areas at the same time. Health education for farm workers from other areas and indigenous people should be enhanced. It is essential that farmworkers dress in light color clothes and check whether they have been bitten by ticks[24]. In addition, a quarantine should be enforced for the border-crossing transportation and trade of domestic animals, especially to check whether the animals carry ticks.

Several limitations should be noted within our study. First, China did not enforce testing of SFTS until now, and the cases in this study were collected from a passive surveillance system. The reporting level should be considered in future studies. Mild and subclinical cases that were not treated with medical care may also be identified and used to correct the epidemiological characteristics of this disease. Second, the level of diagnosis and detection among the counties was different. The situation in some counties reporting fewer cases may not represent the real epidemic. Therefore, the heterogeneity of the component driving the transmission of SFTS across counties would be biased. Third, the data regarding risk factors, including land cover, pathogen dynamics, tick density and animal infection rate, were not collected. The correlation between the climate factors and the incidence of SFTS would thus be overestimated. Future studies should incorporate these cofactors into the model to achieve a more accurate estimation.

In conclusion, Zhejiang Province has a high case fatality rate and a high incidence of SFTS. Furthermore, the numbers of affected counties has been increasing year by year, and the interval from illness onset to confirmation of SFTS cases is too long. Spatial heterogeneity in the autoregressive and spatiotemporal components drove the transmission of SFTS according to the multivariate time series model. In addition, the ranges of suitable weather values for the transmission of SFTS was identified. This suggests that targeted preventive efforts should be made in different areas based on the main component contributing to the epidemics. In particular, early measures several months ahead of the suitable season for the occurrence of SFTS should be implemented. Enhancement of the level of reporting and diagnosis of this disease should strengthen ongoing research with the incorporation of more risk factors.

## Supporting information

S1 Checklist STROBE checklist. (DOCX)

S1 File The database of key information of SFTS cases. (XLSX)

S1 Fig Map of Zhejiang Province, China with area names. This map was created by ArcGIS software (version 10.1, ESRI Inc.; Redlands, CA, USA). The homepage for the ArcGIS software was https://www.esri.com/.
(TIF)
