# Supplementary figures and images for: Spatial-temporal characteristics of severe fever with thrombocytopenia syndrome and the relationship with meteorological factors from 2011 to 2018 in Zhejiang Province, China
Source: PLoS Negl Trop Dis. 2020 Apr 7;14(4):e0008186. doi: 10.1371/journal.pntd.0008186 (PMC7164674; doi:10.1371/journal.pntd.0008186)

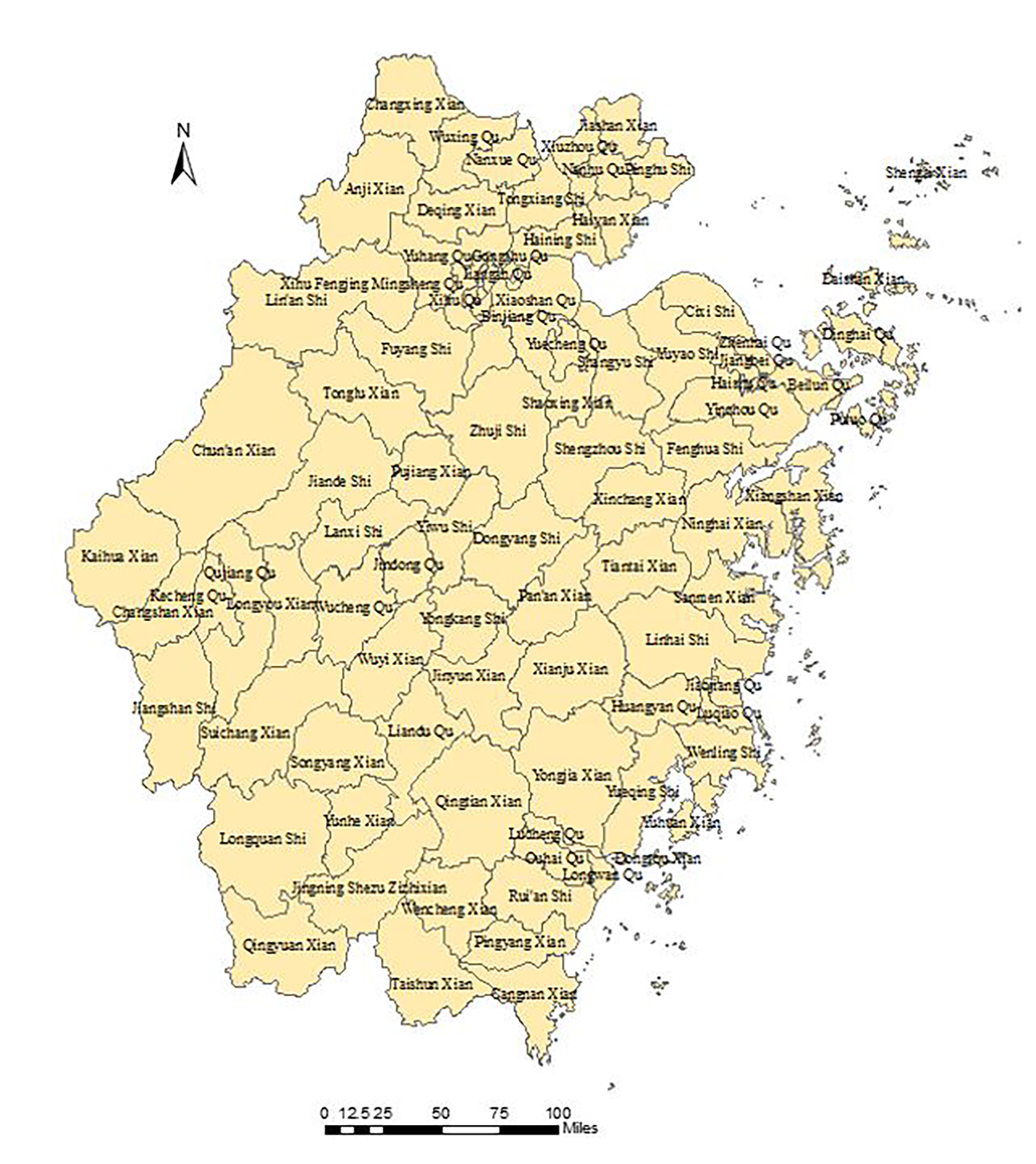

Supplement: S1 Fig — This map was created by ArcGIS software (version 10.1, ESRI Inc.; Redlands, CA, USA). The homepage for the ArcGIS software was https://www.esri.com/. (TIF) [file pntd.0008186.s003.tif]
